# Identification of novel B cell epitopes in the fiber protein of serotype 8 Fowl adenovirus

**DOI:** 10.1186/s13568-019-0895-1

**Published:** 2019-10-31

**Authors:** Hao Lu, Hongxia Shao, Hongjun Chen, Jianjun Zhang, Weikang Wang, Tuofan Li, Quan Xie, Aijian Qin, Jianqiang Ye

**Affiliations:** 1 Key Laboratory of Jiangsu Preventive Veterinary Medicine, Key Laboratory for Avian Preventive Medicine, Ministry of Education, College of Veterinary Medicine, Yangzhou University, Yangzhou, 225009 Jiangsu China; 2 Jiangsu Co-innovation Center for Prevention and Control of Important Animal Infectious Diseases and Zoonoses, Yangzhou, 225009 Jiangsu China; 3 Joint International Research Laboratory of Agriculture and Agri-Product Safety, The Ministry of Education of China, Yangzhou University, Yangzhou, 225009 China; 4 Institutes of Agricultural Science and Technology Development, Yangzhou University, Yangzhou, 225009 Jiangsu China; 5 Shanghai Veterinary Research Institute, Chinese Academy of Agricultural Sciences, 518 Ziyue Road, Shanghai, 200241 China; 6 Sinopharm Yangzhou VAC Biological Engineering Co. Ltd, Yangzhou, 225127 Jiangsu China

**Keywords:** FAdV-8, Fiber protein, Monoclonal antibodies, Reactivity, Epitope

## Abstract

In recent years, hepatitis-hydropericardium syndrome (HHS) and inclusion body hepatitis (IBH) caused by fowl adenovirus (FAdV) infection have resulted in significant economic losses to the poultry industry worldwide. Epidemiological analysis revealed that serotype FAdV-8 is one of the major pathogenic FAdVs currently prevalent in domestic flocks. Although the fiber protein of FAdV plays vital roles in viral infection and pathogenesis, the B cell epitope in the fiber protein is less known. In this study, two monoclonal antibodies (mAbs) specific to fiber protein of FAdV-8, designated as 4D9 and 5F10, were prepared. Although the mAb 4D9 and 5F10 could not neutralize FAdV-8 infection, 4D9 and 5F10 showed good activities of indirect immunofluorescence, western blot and immunoprecipitation. Epitope analysis revealed that mAb 5F10 recognized 187–219aa in the fiber whereas mAb 4D9 recognized 113–149aa in the fiber. Sequence analysis showed that the epitope recognized by mAb 5F10 was conserve across serotypes FAdV-7, 8a and 8b whereas that for mAb 4D9 was only conserve in FAdV-8b. The generation of mAbs specific to fiber of FAdV-8 and the identification of the novel B cell epitopes here lay the foundation for further studying the antigenicity of the fiber and developing specific diagnosis for FAdV-8.

## Introduction

Fowl adenovirus (FAdV) is currently clustered into five species (A–E) and twelve serotypes (1–7, 8a/b, 9–11) (Hess 2000; Schachner et al. 2014; Takeuchi et al. 1999; Meulemans et al. 2001). Most of these serotypes FAdVs generally cause subclinical symptoms in the infected chickens, whereas the infection of several serotypes of FAdVs result in inclusion body hepatitis (IBH), hepatitis-hydropericardium syndrom (HHS), adenoviral gizzard erosion (AGE) (Mittal et al. 2014; Niczyporuk 2016; Okuda et al. 2004). Serotypes FAdV-2, FAdV-8 and FAdV-11 generally induce IBH whereas FAdV-4 is the major causative agent for HHS, and FAdV-1 is related to AGE (Schachner 2018; Grgic et al. 2014). Recently, the outbreaks of IBH and HHS caused by FAdV-8 and FAdV-4, respectively have resulted in huge economic loss in poultry industry worldwide (Schachner 2018; Grgic et al. 2014). However, the molecular pathogenesis of the highly pathogenic FAdV is less known, and the vital antigens or antigenic sites for the development of efficient vaccines or specific diagnosis need to be determined for better controlling the diseases of IBH and HHS. Several studies have shown that the fibers of FAdV-4 play vital role in mediating virus infection and induces immuno-responses in the infected chickens (Schachner 2014; Ruan 2018; Chen 2018; Wang et al. 2018). Notably, serotypes of FAdV-1, 4, 10 have two fiber proteins whereas the other serotypes of FAdVs including FAdV-8 have only one fiber protein. However, the mechanism that the serotype FAdV-1, 4, 10 have two fibers whereas others only have one need to be further elucidated. In this study, we used the purified prokaryotic fusion protein GST-fiber as immunogen to generate two mAbs specific to the fiber protein of FAdV-8, and identified two novel B cell epitopes in the fiber protein.

## Materials and methods

### Viruses, cells, plasmids and proteins

FAdV-1 (ATCC^®^VR-432™), FAdV-4 (ATCC^®^VR-829™), FAdV-5 (ATCC^®^VR-830™), FAdV-6 (ATCC^®^VR-831™), FAdV-7 (ATCC^®^VR-832™), FAdV-9 (ATCC^®^VR-833™) and FAdV-10 (ATCC^®^VR-834™) were from ATCC and FAdV-8 isolate JSSQ15 (FAdV-8b) (Ye et al. 2016) was isolated and maintained in our laboratory. The LMH cell was cultured in F12/DMEM (Gibco, NY, USA) with 10% FBS (Lonsera, Shanghai, China). Plasmids pcDNA3.1 and pGEX-6P-1 vector were stored in our laboratory. Plasmid pcDNA3.1-F expressing the Fiber protein of FAdV-8b was prepared and stored in our laboratory. The purified prokaryotic fusion protein GST-fiber of FAdV-8 was prepared in our laboratory.

### Antibodies

Monoclonal antibody against the hexon of FAdV (mAb 1B4) was a kindly gift from Prof. Hongjun Chen. Monoclonal antibody against NP of avian influenza virus (mAb 4A7) was prepared and stored in our laboratory. FITC-labelled goat anti-mouse IgG and HRP-labelled goat anti-mouse IgG were from Sigma (CA, USA).

### Generation of mAbs

6-week-old BALB/C mice were immunized with 50 μg of the purified GST-fiber four times every 7 days. At day 3 following the fourth immunization, the spleen cells from one immunized mouse were fused with SP2/0 cells (Roche, Mannheim, Germany), as previously described (Nelson et al. 2000). The hybridoma cells secreting antibodies against the fiber protein of FAdV-8 were screened through immunofluorescence assay (IFA) using the cells infected with FAdV-8. The positive hybridoma cells were subcloned, and the characteristics of mAbs secreted by these positive clones were identified through western blot, IFA and immunoprecipitation. The isotype of mAb was determined with a mouse mAb isotyping kit (Thermo Scientific, Massachusetts, USA) according to the manufacturer’s protocol. The ascites of these mAbs were generated and purified as previously described (Ye 2010).

### Indirect immunofluorescence assay (IFA) and western blot

To identify the characteristics of mAbs, LMH cells infected with 0.01 MOI of FAdV-8 JSSQ15 or transfected with pcDNA3.1-F were assessed using mAbs by IFA and western blot. For IFA, the LMH cells at 3 days post-infection or transfection were fixed with the chilled acetone and ethanol (3:2) for 5 min. mAbs at the indicated dilutions were incubated with the fixed cells for 45 min. After three washes with PBS, FITC-conjugated secondary antibodies at 1:200 dilutions were reacted with the cells for another 45 min. After three washes with PBS, the cell imaging was observed on an inverted fluorescence microscope. For western blot, the LMH cells at 3 days post-infection or transfection were collected and lysed in lysis buffer (CST, MA, USA) with PMSF (Beyotime, Shanghai China), protease and phosphatase inhibitors (CST). The lysed cell supernatants were boiled with the loading buffer. After separation via SDS-PAGE, the denatured samples were transferred onto nitrocellulose membranes (NCs) (GE Healthcare Life sciences, Freiburg, Germany). After blocked with 5% skim milk in PBST for 1 h at room temperature (RT), the NCs were reacted with mAbs (1:1000 dilution in 5% skim milk) at 4 °C overnight. After three washes with PBST, the NCs were incubated with HRP-labelled goat anti-mouse IgG (1:10 000 dilution in 5% skim milk). After another three washes, the NCs were developed using an automatic chemiluminescence image analysis system (Tanon 5200) (Wang et al. 2018).

### Immunoprecipitation (IP)

LMH cells infected with 0.01 MOI of FAdV-8 at 3 dpi were collected and lysed in lysis buffer (CST, MA, USA) with PMSF (Beyotime, Shanghai China), protease and phosphatase inhibitors (CST). Lysates were then mixed and immunoprecipitated with 2 μg of mAb at 4 °C overnight. 25 μL of protein G-Sepharose beads (Beyo-time) were added to the mixture and incubated at 4 °C for 2.5 h. Then, the mixture of protein and bead was washed five times with PBS, and the immunoprecipitated proteins were analyzed by western blot.

### Epitope mapping

To map the epitopes recognized by mAbs, the different truncated fiber genes derived from FAdV-8 were constructed with a ClonExpress II One Step Cloning Kit (Vazyme Biotech, Nanjing, China). The primers used for amplifying the different segment of fiber gene, the linear pGEX-6P-1 vector and pcDNA3.1 vector were listed in Table 1. After confirmed by sequencing, the recombinants were transformed into *E. coli* BL21 or transfected into LMH cells for the expression. IFA and western blot were used to identify the epitopes of mAbs using the constructed recombinants.

**Table 1:** Primers for amplifying the truncated Fiber gene, the linear pcDNA3.1 and pGEX-6p-1

PCR product	Primer sequence (5′–3′)
pG-F-1–186aa	Forward: GTTCCAGGGGCCCATGGCGACCTCGACTCC
	Reverse: GTTTTCACCGTCATTAGGTCTCATCGACTCT
pG-F-187–360aa	Forward: GTTCCAGGGGCCCATGTTGCTCATCGAGGATGA
	Reverse: GTTTTCACCGTCATTAGAGCTTCAGATAGAGGG
pG-F-361–519aa	Forward: GTTCCAGGGGCCCATGGACAGAGCACAGTTCAC
	Reverse: GTTTTCACCGTCATTAAGGAGCGTTGGCGG
pc-F-1–186aa	Forward: AGCTTGGTACCGAATGGCGACCTCGACTCC
	Reverse: ATATCTGCAGAATTTAGGTCTCATCGACTCT
pc-F-1–149aa	Forward: AGCTTGGTACCGAATGGCGACCTCGACTCC
	Reverse: ATATCTGCAGAATTTAAGGGTCGACCGACAG
pc-F-1–112aa	Forward: AGCTTGGTACCGAATGGCGACCTCGACTCC
	Reverse: ATATCTGCAGAATTTAGGCGAGTGTCAGGGC
pc-F-187–360aa	Forward: AGCTTGGTACCGAATGTTGCTCATCGAGGATGA
	Reverse: ATATCTGCAGAATTTAGAGCTTCAGATAGAGGG
pc-F-187–287aa	Forward: AGCTTGGTACCGAATGTTGCTCATCGAGGATGA
	Reverse: ATATCTGCAGAATTTAGAGTGTGAGCCCGGT
pc-F-187–250aa	Forward: AGCTTGGTACCGAATGTTGCTCATCGAGGATGA
	Reverse: ATATCTGCAGAATTTAAGTCTGTACGGTAAG
pG-F-113–149aa	Forward: GTTCCAGGGGCCCATGTATGAACCGGAGAGTCTCGAG
	Reverse: GTTTTCACCGTCATTAAGGGTCGACCGACAGCTGTAT
pG-F-187–219aa	Forward: GTTCCAGGGGCCCATGATGTTGCTCATCGAGGATGA
	Reverse: GTTTTCACCGTCATTAGTCCAGGCCCTGTTCGTCG
Linear pcDNA3.1	Forward: GAATTCTGCAGATATCCAGCACAGTG
	Reverse: GCTCGGTACCAAGCTTAAGTTTAAACG
Linear pGEX-6P-1	Forward: TAATGACGGTGAAAACCTCTGACACATGC
	Reverse: CATGGGCCCCTGGAACAGAACTTCCAGAT

### Sequence analysis of epitopes on the Fiber of species FAdV-E

The Fiber proteins of FAdV-E (including serotype FAdV-6, 7, 8a and 8b) with the identified epitopes were compared and aligned using MegAlign software (DNASTAR, Madison, WI, USA).

## Results

### Two novel mAbs against fiber protein of FAdV-8 were developed

**Fig. 1:**
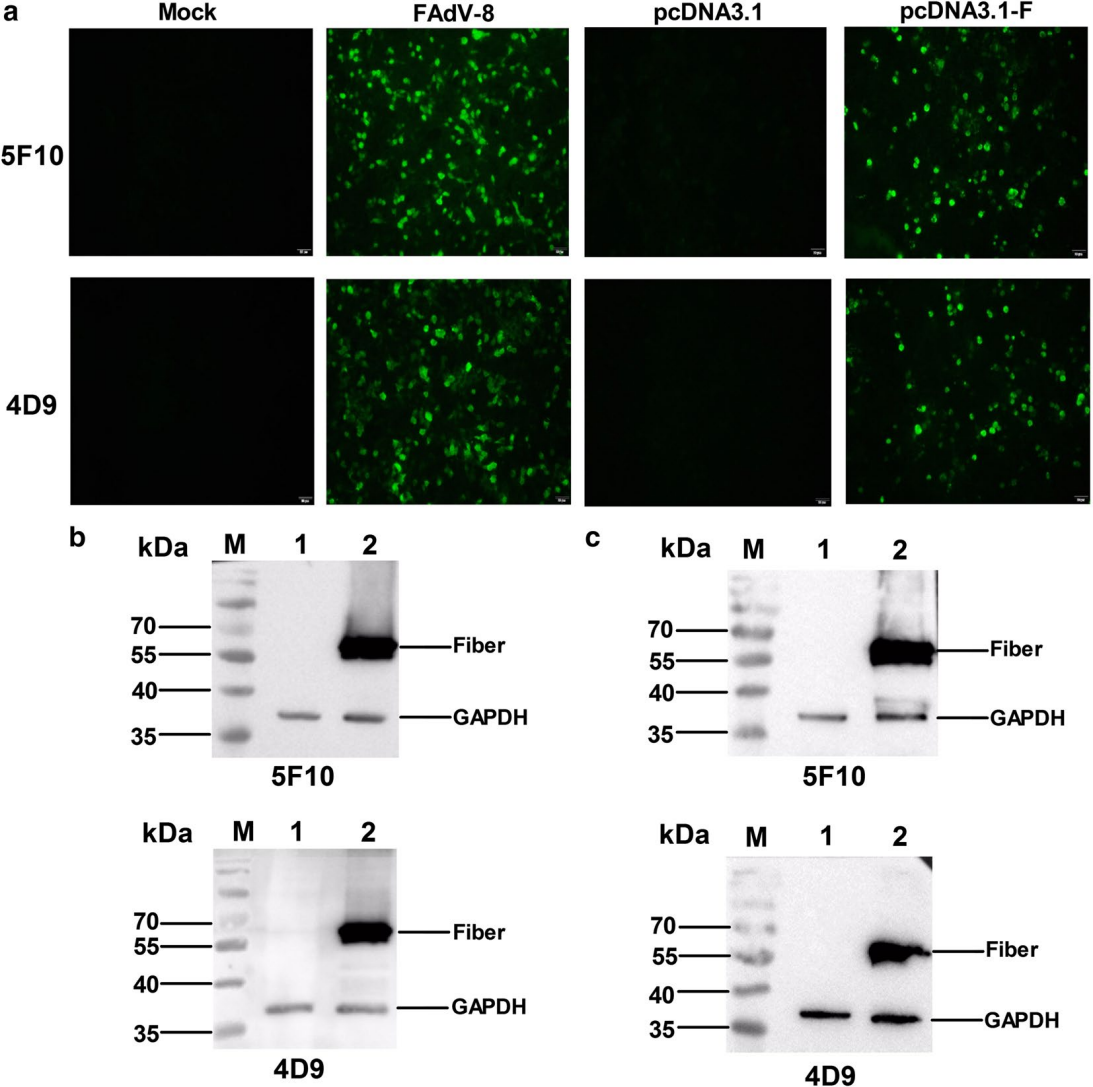
Identification of two mAbs 5F10 and 4D9 against fiber protein of FAdV-8. LMH cells infected with 0.01 MOI of FAdV-8 or transfected with pcDNA3.1-F were analyzed using mAbs by IFA and western blot. **a** The LMH cells infected with FAdV-8 or transfected with pcDNA3.1-F were analyzed using mAbs by IFA. The uninfected LMH cells and the LMH cells transfected with pcDNA3.1 were as negative control. **b** The lysates of LMH cells infected with FAdV-8 were analyzed using mAbs by western blot. Lanes 1 and 2: lysates of LMH cells infected without or with FAdV-8, respectively. **c** The lysates of LMH cells transfected with pcDNA3.1-F were analyzed using mAbs by western blot. Lanes 1 and 2: lysates of LMH cells transfected with pcDNA3.1 and pcDNA3.1-F, respectively

To generate mAbs against fiber protein of FAdV-8, Balb/c mice were immunized with the purified GST fusion protein GST-fiber and the LMH cells infected with FAdV-8 were used as a screening antigen. Finally, two mAbs, designated as 4D9 and 5F10, showed strong positive reaction with the LMH cells infected with FAdV-8 or transfected with pcDNA3.1-fiber in IFA as shown in Fig. 1a. Moreover, mAbs 4D9 and 5F10 could efficiently recognize the linear epitopes of the fiber protein expressed in the infected or transfected LMH cells in western blot as described in Fig. 1b, c. The subtype of the two mAbs 4D9 and 5F10 was IgG2a and IgG1, respectively identified using a mouse mAb isotyping kit (Thermo) according to the manufacturer’s protocol. The mAbs in ascites were prepared and purified using protein G columns (GE Healthcare Life science).

### 4D9 and 5F10 could efficiently immunoprecipitate the fiber protein of FAdV-8

**Fig. 2:**
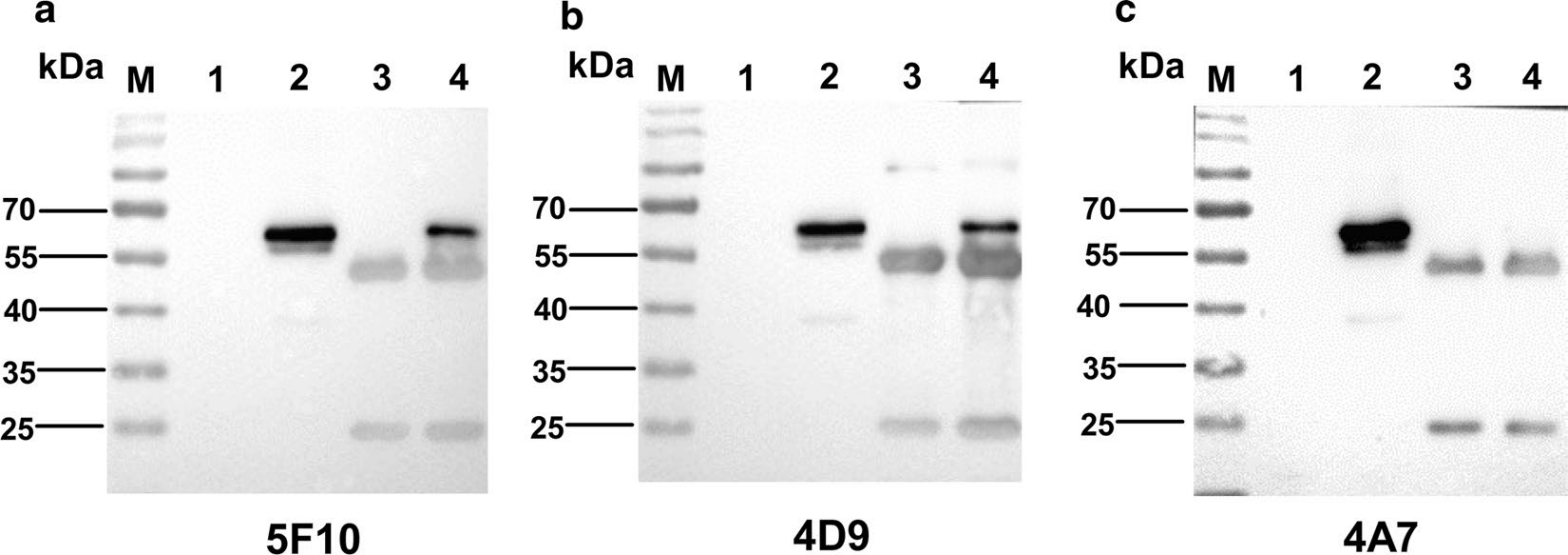
Immunoprecipitation analysis for mAbs 5F10 and 4D9. LMH cells infected with 0.01 MOI of FAdV-8 were analyzed using mAbs by immunoprecipitation assay, followed by western blot analysis using a mice polyclonal antibody. **a** Lanes 1 and 2: lysates of LMH cells infected without or with FAdV-8, respectively. Lanes 3 and 4: mAb 5F10 immunoprecipitated pellets of LMH cells infected without or with FAdV-8, respectively. **b** Lanes 1 and 2: lysates of LMH cells infected without or with FAdV-8, respectively. Lanes 3 and 4: mAb 4D9 immunoprecipitated pellets of LMH cells infected without or with FAdV-8, respectively. **c** Lanes 1 and 2: lysates of LMH cells infected without or with FAdV-8, respectively. Lanes 3 and 4: mAb 4A7 immunoprecipitated pellets of LMH cells infected without or with FAdV-8, respectively

To evaluate whether mAbs 4D9 and 5F10 could immunoprecipitate the fiber protein in cells infected with FAdV-8, the immunoprecipitation assay was performed by using mAbs 4D9 and 5F10. As described in Fig. 2, a specific band of fiber protein with the size of 57kD in the lysates from LMH cells infected with FAdV-8 could be immunoprecipitated by mAbs 4D9 and 5F10, respectively whereas such protein band could not be immunoprecipitated by the control mAb 4A7. This data demonstrated that the mAbs 5F10 and 4D9 could efficiently immunoprecipitate the fiber protein in LMH cells infected with FAdV-8, highlighting that the two mAbs developed here can be used to identifying the host protein or cell receptor for the binding to the fiber protein of FAdV-8 through the co-immunoprecipitation assay.

### The reaction profile for the two mAbs 4D9 and 5F10

**Fig. 3:**
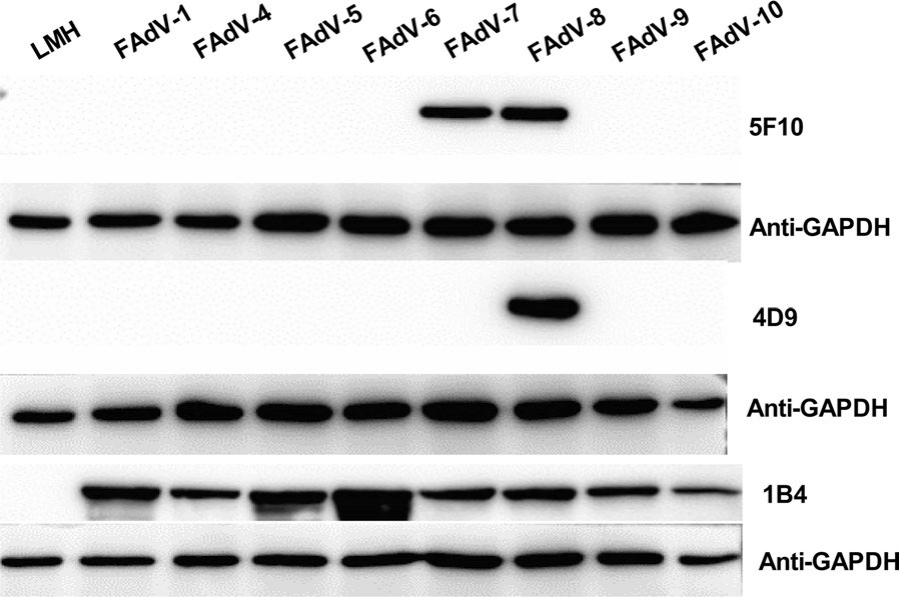
Reaction profile of the mAbs 5F10 and 4D9 analyzed by western blot. The LMH cells infected with FAdV-1, 4, 5, 6, 7, 8, 9, 10 at 0.01 MOI, respectively were analyzed using mAbs 5F10 and 4D9 by western blot. The uninfected LMH cells were as negative controls, mAb against hexon was as positive control, and the GAPDH was as protein loading control

To investigate the reaction profile for the two mAbs 4D9 and 5F10, LMH cells infected with different serotypes of fowl adenoviruses including FAdV-1, FAdV-4, FAdV-5, FAdV-6, FAdV-7, FAdV-8, FAdV-9 and FAdV-10 were analyzed using mAbs 4D9, 5F10 and 1B4 by the western blot. As shown in Fig. 3, mAb 4D9 only reacted with the fiber protein of FAdV-8, not with the fiber protein from other FAdVs tested whereas mAb 5F10 could recognize the fiber protein from both FAdV-7 and FAdV-8. Different from mAbs 4D9 and 5F10, mAb 1B4 against hexon could efficiently recognize the hexon protein in the LMH cells infected with FAdV-1, FAdV-4, FAdV-5, FAdV-6, FAdV-7, FAdV-8, FAdV-9 and FAdV-10, respectively, but not reacted with the control LMH cells. All these data demonstrate that mAb 4D9 is highly specific to the fiber protein of FAdV-8 whereas mAb 5F10 recognizes the common epitope in the fiber proteins between FAdV-7 and FAdV-8.

### Mapping of epitope in fiber protein recognized by mAb 5F10 and 4D9

**Fig. 4:**
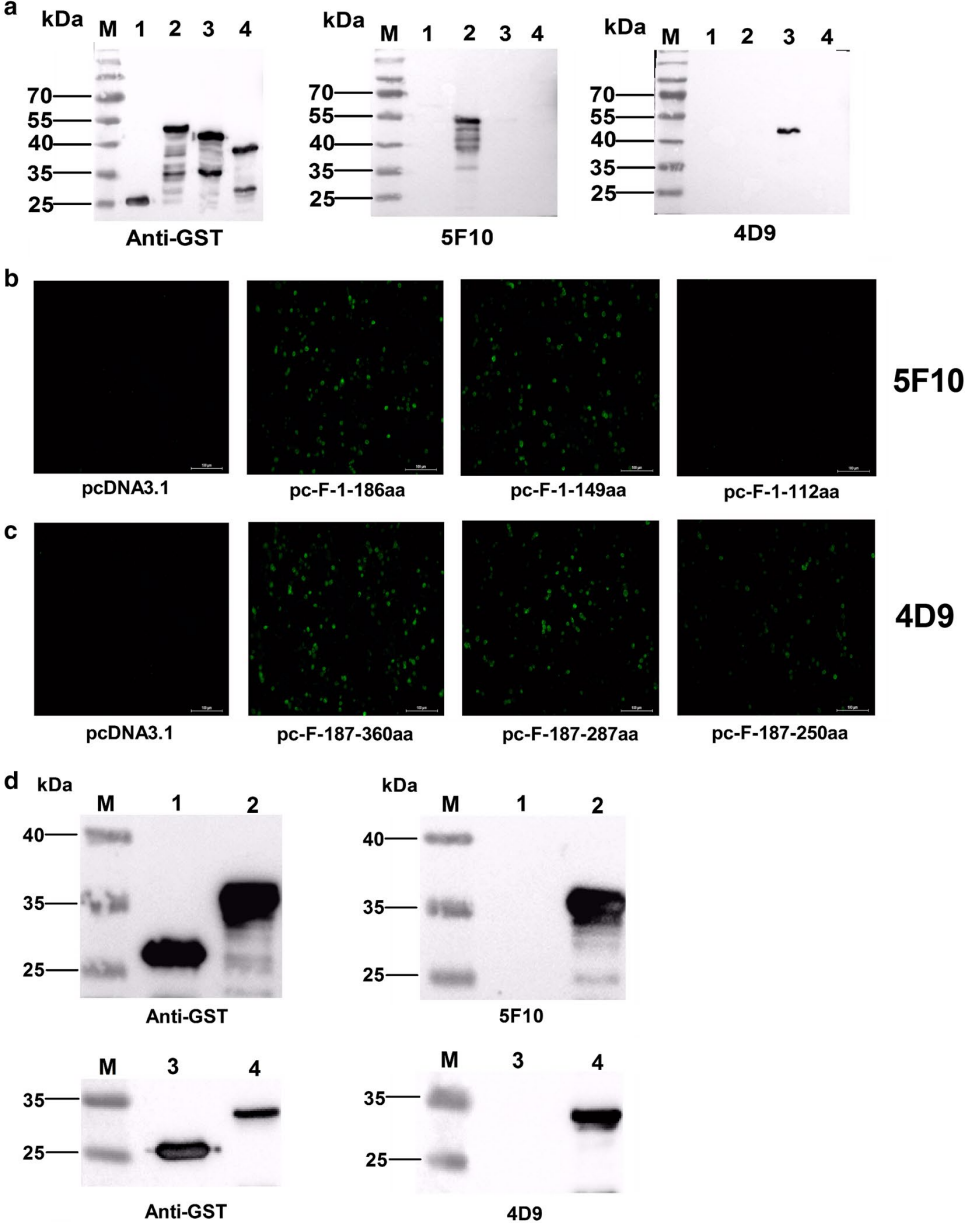
Mapping of B cell epitopes recognized by mAbs 5F10 and 4D9. **a** The different truncated GST-fiber fusion proteins were analyzed using mAbs 5F10 and 4D9 by western blot. The mAb against GST was as positive control. Lane 1, 2, 3 and 4: the lysate of the IPTG induced BL21 cells transformed with pGEX-6p-1, GST-F-1–186aa, GST-F-187–360aa and GST-F-361–519aa, respectively. **b** The LMH cells transfected with the different truncated fiber genes F-1–186aa, F-1–149aa, F-1–112aa and the control plasmid pcDNA3.1, respectively were analyzed using mAb 5F10 by IFA. **c** The LMH cells transfected with the different truncated fiber genes F-187–360aa, F-187–287aa, F-187–250aa and the control plasmid pcDNA3.1, respectively were analyzed using mAb 4D9 by IFA. **d** The different truncated GST-fiber fusion proteins were analyzed using mAbs 5F10 and 4D9 by western blot. The mAb against GST was as positive control. Lane 1, 2, 3 and 4: the lysate of the IPTG induced BL21 cells transformed with pGEX-6p-1, GST-F-113–149aa, pGEX-6p-1 and GST-F-187–219aa, respectively

**Fig. 5:**
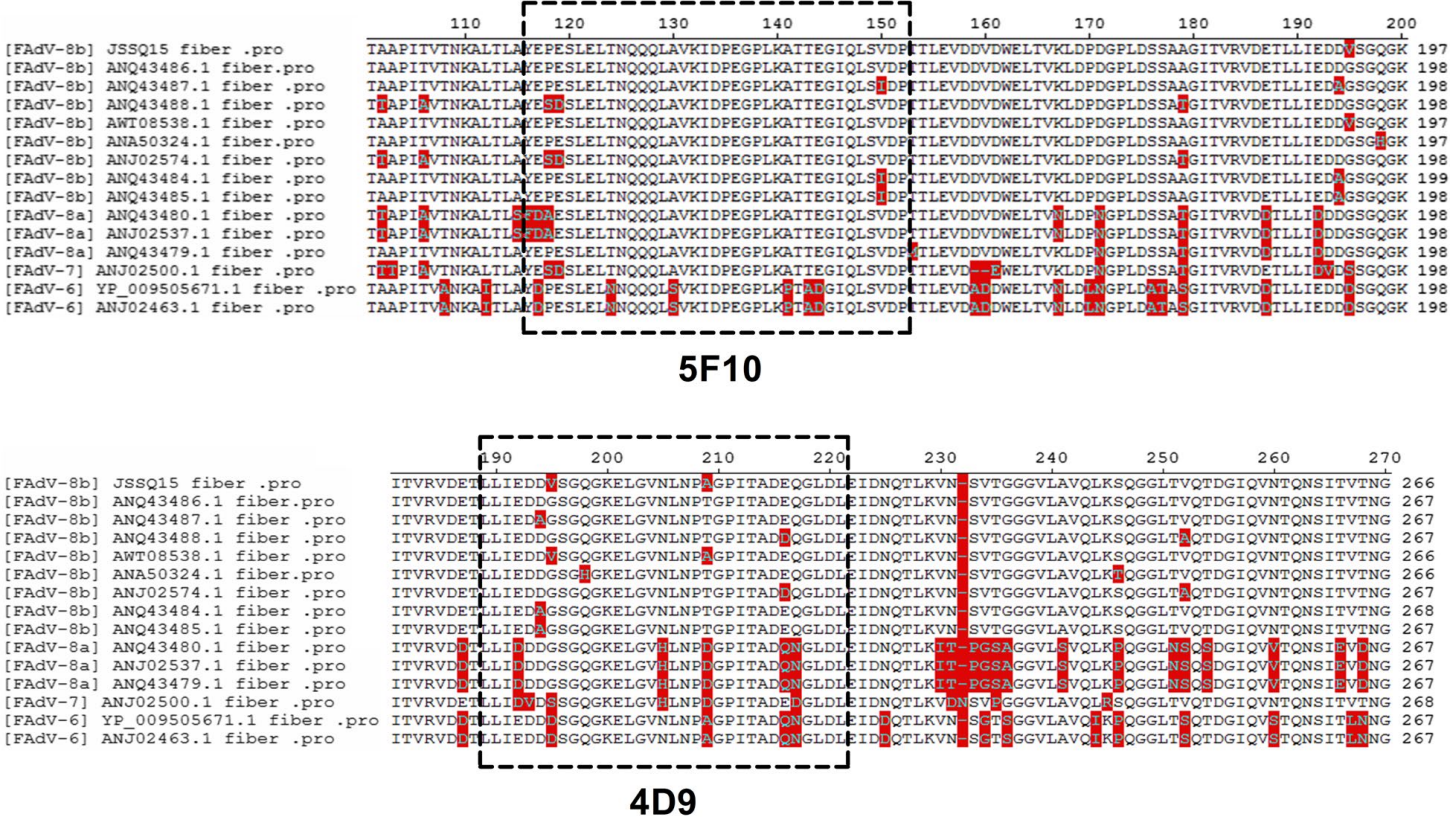
Analysis of the identified epitopes recognized by mAbs 5F10 and 4D9 The Fiber proteins of serotype FAdV-6, 7, 8a and 8b were aligned and the epitopes recognized by mAbs 5F10 and 4D9 were analyzed using DNAStar software. The epitope recognized by mAb 5F10 was conserve across species FAdV-7, 8a and 8b whereas the region recognized by mAb 4D9 was only conserve in FAdV-8b

To determine the epitopes recognized by the two novel mAbs 4D9 and 5F10, three truncated Fiber (F-1–186aa, F-187–360aa and F-361–519aa) fused with GST were first constructed and expressed in *E. coli.* BL21. As described in Fig. 4a, 5F10 recognized F-1–186aa whereas 4D9 recognized F-187–360aa. To refine the epitope recognized by mAbs 4D9 and 5F10, a serial of truncated Fibers with deletions at the C-terminus were constructed and expressed in LMH cells and *E. coli*. As shown in Fig. 4b–d the epitope recognized by mAb 4D9 located in 187–219aa and that for 5F10 was in 113–149aa. To evaluate the variation of the two identified epitopes among species FAdV-E, the Fiber proteins of serotype FAdV-6, 7, 8a and 8b were aligned by using MegAlign software. As shown in Fig. 5, the epitope of 5F10 was highly conserve across species FAdV-7, FAdV-8a and FAdV-8b. However, the epitope recognized by 4D9 was only conserve in serotype FAdV-8b, but not in FAdV-7 and FAdV-8a.

## Discussion

Hexon, penton and fiber are the major structural proteins encoded by FAdV. Although hexon, penton and fiber all contain dominant antigenic sites, hexon and penton proteins are certain conserved among different serotypes of FAdV whereas fiber is various in different FAdVs. Therefore, fiber protein is a suitable target for developing serotype specific diagnosis and vaccines for FAdV. Fiber based ELISAs for detection of the antibody or antigen of FAdV-4 have been reported (Shao et al. 2019; Feichtner et al. 2017; He et al. 2018). Several groups reported that fiber based subunit vaccines of FAdV-4 were better than hexon based vaccines (Schachner et al. 2014). More recently, a monoclonal antibody 3C2 against the C-terminus of the fiber2 was reported to have efficient neutralizing activity for the infection of FAdV-4 in vitro (Wang et al. 2018). Although FAdV-8 is an important causative agent for chicken inclusion body hepatitis, the Fiber protein of FAdV-8 is less studied. In this study, two novel mAbs 4D9 and 5F10 against fiber of FAdV-8 were prepared and their epitopes were uncovered. mAbs 4D9 and 5F10 recognize 187–219aa and 113–149aa in the fiber of FAdV-8, respectively. Although mAbs 4D9 and 5F10 did not show neutralizing activity against the infection of FAdV-8, they had IFA, western blot and IP activity for the fiber protein of FAdV-8. Specificity analysis revealed that mAb 4D9 was highly specific to FAdV-8 whereas mAb 5F10 showed cross-reaction with FAdV-7, but not with other serotypes tested. This data is also consistent with the fact that the Fiber among different species of FAdV (A-E) shows low identity whereas that in the same species has certain high homology. Amino acid sequence assay also confirmed this reaction profile of the two novel mAbs as described in Fig. 5.

Summary, this is the first identification of two novel B cell epitopes 187–219aa and 113–149aa in the fiber of FAdV-8 recognized by mAb 4D9 and 5F10, respectively. Whether the two mAbs generated and the two epitopes identified can be used to developing epitope based ELISA for detection of antibody against FAdV-8 and sandwich ELISA for detection of antigen of FAdV-8 need to be further investigated. In addition, the efficient immunoprecipitation activity for the two mAbs highlighted their application in identifying the host proteins interacting with the fiber and then to further elucidate the roles of fiber in the pathogenesis of FAdV-8.

## Data Availability

The datasets used and/or analysed during the current study are available from the corresponding author on reasonable request.

## References

[CR1] Chen L (2018). Immunogenicity and protective efficacy of recombinant fiber-2 protein in protecting SPF chickens against fowl adenovirus 4. Vaccine.

[CR2] Feichtner F (2017). Development of sensitive indirect enzyme-linked immunosorbent assays for specific detection of antibodies against fowl adenovirus serotypes 1 and 4 in chickens. Avian Pathol.

[CR3] Grgic H, Krell PJ, Nagy E (2014). Comparison of fiber gene sequences of inclusion body hepatitis (IBH) and non-IBH strains of serotype 8 and 11 fowl adenoviruses. Virus Genes.

[CR4] He ZR (2018). Recombinant fiber-2 protein-based indirect ELISA for antibody detection of fowl adenovirus serotype 4. Avian Dis.

[CR5] Hess MJAP (2000). Detection and differentiation of avian adenoviruses: a review. Avian Pathol.

[CR6] Meulemans G (2001). Polymerase chain reaction combined with restriction enzyme analysis for detection and differentiation of fowl adenoviruses. Avian Pathol.

[CR7] Mittal D (2014). Characterization of fowl adenoviruses associated with hydropericardium syndrome and inclusion body hepatitis in broiler chickens.. Virusdisease.

[CR8] Nelson PN (2000). Monoclonal antibodies. Mol Pathol.

[CR9] Niczyporuk JS (2016). Phylogenetic and geographic analysis of fowl adenovirus field strains isolated from poultry in Poland. Arch Virol.

[CR10] Okuda Y (2004). Pathogenicity of serotype 8 fowl adenovirus isolated from Gizzard erosions of slaughtered broiler chickens. J Vet Med..

[CR11] Ruan S (2018). A subunit vaccine based on fiber-2 protein provides full protection against fowl adenovirus serotype 4 and induces quicker and stronger immune responses than an inactivated oil-emulsion vaccine. Genet Evol..

[CR12] Schachner A (2014). Recombinant FAdV-4 fiber-2 protein protects chickens against hepatitis-hydropericardium syndrome (HHS). Vaccine.

[CR13] Schachner A (2018). Fowl adenovirus-induced diseases and strategies for their control—a review on the current global situation. Avian Pathol..

[CR14] Shao H (2019). A novel monoclonal antibodies-based sandwich ELISA for detection of serotype 4 fowl adenovirus. Avian Pathol.

[CR15] Takeuchi S (1999). Serotyping of adenoviruses on conjunctival scrapings by PCR and sequence analysis. J Clin Microbiol.

[CR16] Wang P (2018). A novel monoclonal antibody efficiently blocks the infection of serotype 4 fowl adenovirus by targeting fiber-2. Vet Res..

[CR17] Ye JQ (2010). Intranasal delivery of an IgA monoclonal antibody effective against sublethal H5N1 influenza virus infection in mice. Clin Vacc Immunol..

[CR18] Ye J (2016). Outbreaks of serotype 4 fowl adenovirus with novel genotype, China. Emerg Microb Infect.

